# Fisheries management as a Stackelberg Evolutionary Game: Finding an evolutionarily enlightened strategy

**DOI:** 10.1371/journal.pone.0245255

**Published:** 2021-01-20

**Authors:** Monica Salvioli, Johan Dubbeldam, Kateřina Staňková, Joel S. Brown

**Affiliations:** 1 Department of Mathematics, Politecnico di Milano, Milano, Italy; 2 Department of Mathematics, University of Trento, Trento, Italy; 3 Department of Data Science and Knowledge Engineering, Maastricht University, Maastricht, The Netherlands; 4 Delft Institute of Applied Mathematics, Delft University of Technology, Delft, The Netherlands; 5 Department of Integrated Mathematical Oncology, Moffitt Cancer Center, Tampa, FL, United States of America; University of Electronic Science and Technology of China, CHINA

## Abstract

Fish populations subject to heavy exploitation are expected to evolve over time smaller average body sizes. We introduce Stackelberg evolutionary game theory to show how fisheries management should be adjusted to mitigate the potential negative effects of such evolutionary changes. We present the game of a fisheries manager versus a fish population, where the former adjusts the harvesting rate and the net size to maximize profit, while the latter responds by evolving the size at maturation to maximize the fitness. We analyze three strategies: i) ecologically enlightened (leading to a Nash equilibrium in game-theoretic terms); ii) evolutionarily enlightened (leading to a Stackelberg equilibrium) and iii) domestication (leading to team optimum) and the corresponding outcomes for both the fisheries manager and the fish. Domestication results in the largest size for the fish and the highest profit for the manager. With the Nash approach the manager tends to adopt a high harvesting rate and a small net size that eventually leads to smaller fish. With the Stackelberg approach the manager selects a bigger net size and scales back the harvesting rate, which lead to a bigger fish size and a higher profit. Overall, our results encourage managers to take the fish evolutionary dynamics into account. Moreover, we advocate for the use of Stackelberg evolutionary game theory as a tool for providing insights into the eco-evolutionary consequences of exploiting evolving resources.

## 1 Introduction

### 1.1 Fisheries-induced evolution

Both freshwater and saltwater fisheries are experiencing extraordinary mortality increase with respect to known historic levels and the primary cause of this increased mortality is fishing [1–4]. Overfishing reduces the recovery rate of populations and species and can lead to extinction [2]. Additionally, intense and prolonged harvest may cause genetic changes in fish, often referred to as “fisheries-induced evolution” [5–9]. The most significant are temporal changes in the life-history traits of the exploited stocks, including altered investments into growth, maturation and reproduction. In particular, fishing generally selects for earlier maturation at smaller sizes. The mean maturation age for North East Arctic Cod, for example, which was 10-11 years in the 1930s, had dropped to approximately 7 years by the 1990s [10]. Fisheries-induced evolution is likely to be strong and can occur rapidly in intensively exploited populations. Furthermore, the changes in size and maturation age may also be hard to reverse [1, 11, 12].

Intense exploitation reduces the abundance of fish stocks and, due to the smaller size of remaining fish, it decreases fisheries profit. For these reasons, quantifying and predicting the evolutionary response of exploited populations is important for both ecological and economic reasons, in order to develop a harvesting management aimed at mitigating the effects of the evolutionary changes and ensuring a sustainable exploitation of fishery resources [12, 13]. The very first example of an intervention aimed at promoting sustainable management dates back to the Magna Carta signed in 1215 by King John, who removed the fish-weirs throughout England, which were preventing fish, especially salmon, from returning to their spawning grounds upriver [14]. This decision successfully increased the salmon population. Since then, fisheries managers strive, with more or less success, to maintain fish stocks at an ecologically sustainable level. However, their practices usually do not take evolutionary consequences of their actions into account.

### 1.2 Examples of sustainable fisheries management

The Code of Conduct for Responsible Fisheries adopted by the Food and Agriculture Organization of the United Nations aims at conservation of fish stocks by limiting fishing intensity [15]. This can be achieved, for example, by limiting the total allowable catches, i.e. the tonnage or the number of fish that may be caught from a fishery in a period of time. Alternatively, restrictions can be made on the various components of fishing effort, including number and size of fishing vessels, or the amount of time they are allowed to fish. This is the case of the Faeroers, where an effort management scheme was introduced in 1996, imposing limited days at sea and the closure of some areas in order to preserve the local fish stocks of cod, haddock and saithe [16].

Other common conservation measures are based on size limits, usually allowing fishermen to catch only fish that are larger than a certain size. A typical example includes fishing for bluefin tuna in the United States. In order to give fish a chance to spawn before being caught, fishermen may only catch individuals at least 185 centimeters long [17]. A net size policy can also be implemented indirectly, for example, by imposing the safe release of individuals above or below a certain size. The most striking examples of sustainable fishing concern lobster fisheries in Australia, New England (USA) and the Canadian Maritimes, where catches are abundant despite decades of heavy exploitation, thanks to a management that involves, among others, releasing the smallest and the largest lobsters [18, 19]. When managers can change harvesting rates and net sizes, and the fish evolve countermeasures, the challenges and opportunities for the industry become part of a game between managers and the fish.

### 1.3 Mathematical models

Here we summarize previous theoretical works addressing the problem of fisheries-induced evolution from a co-evolutionary perspective, integrating population dynamics and biological evolution with changing economic strategies. The pioneering work by Law and Grey introduces the concept of an evolutionarily stable optimal harvesting strategy (ESOHS) [11]. If the manager knows that a certain harvesting strategy will drive the stock to a certain evolutionary stable strategy (ESS), the ESOHS of the fishery will be harvesting strategies that maximize yield, given the knowledge of how the fish will evolve. The evolutionary strategy of the fish in this work is the age at maturation, while the strategy of the manager distinguishes between a harvesting rate for the spawner fishery and one for the feeder fishery.

Heino extends the work by Law and Grey by presenting a model that accounts for stochasticity in newborn survival and uncertainty in the estimate of population size [20]. The model considers the age of first reproduction as the evolutionary strategy of fish and the harvesting rate as the strategy of the manager. Heino distinguishes between spawner fishery and feeder fishery and compares the performance of different harvesting strategies when evolutionary change is accounted for. In particular, he studies three harvesting strategies: 1) fixed-quota, where the annual target catch is fixed, 2) constant stock size, where the target stock size after harvesting is kept constant, and 3) constant harvesting rate, where a constant fraction of stock is harvested each year.

Blythe and Stokes introduce a model where a constant fraction of all fish larger than a predefined size are removed per unit of time [21]. In particular, they investigate two contrasting harvesting regimes, one with a lower and one with a higher harvesting rate. Under the low-harvesting-rate regime, larger individuals are favored and there is always a strategy leading to a sustainable yield, corresponding to the ESOHS of Law and Grey. In contrast, under a high-harvesting-rate regime, smaller individuals become predominant and a sustainable yield becomes difficult to reach. Instead of using the Evolutionarily Stable Strategy (ESS) concept, Blythe and Stokes focus on those rare strategies that can invade and replace the resident strategies of a population. In their work, the evolutionary strategy is the size at maturation, while the strategy of the manager is the harvesting rate.

While [11, 20] and [21] did not frame their models in game-theoretic terms explicitly, Brown and Parman were the first ones to define the interaction between the fisheries manager and fish stock as an evolutionary game [22]. In their model, fish may evolve to an ESS in response to harvesting and the manager has two alternative strategies: basing harvesting only on ecological considerations or taking evolutionary considerations into account as well. In the first case, the manager takes the life history characteristics of the fish as given, while in the second they anticipate the response of the fish to harvesting. The comparison of these two scenarios, considering evolution or not, was investigated also by Eikeset et al. and by Zimmermann and Jørgensen, who found that the optimal fishing regimes for the two scenarios do not differ much. However, neither model allows the fisheries manager to respond to evolution, but assumes that the manager selects a strategy that stays the same [23, 24]. Faig is the first one who not only defines the problem in game-theoretic terms but also uses solution concepts derived from game theory [25]. Her work, based on North-East Arctic Cod, compares yield reached by two different managers: a manager who ignores evolution, and a manager who dynamically optimizes his/her strategy by considering how the fish will respond in terms of evolution. In her work, the management strategy consists in setting a harvesting rate and a net size, while the fish evolutionary strategy is the probability of maturation. In particular, she shows how a manager who ignores evolution could achieve a Nash equilibrium, but does not formalize the management strategies implemented by a manager who takes evolution into account. This is because such games have not been formalized yet. Our work aims at filling this gap by applying what we term Stackelberg Evolutionary Games.

### 1.4 Stackelberg Evolutionary Games

We consider a Stackelberg Evolutionary Game between a single fish stock and a single profit-maximizing fishery, expanding upon the model by Brown and Parman [22]. The fish engage in an evolutionary game where ecological dynamics describe changes in population size and evolutionary dynamics describe changes in heritable traits. In an evolutionary game the strategies of the players are inherited, and their payoffs take the form of increased survivorship and/or fecundity [26, 27]. The solutions to this kind of game are evolutionarily stable strategies, strategies that when adopted by an entire population cannot be invaded by any rare alternative strategies. On the other hand, the fisheries manager is involved in a game with fish, which we call a Stackelberg (or leader-follower) Evolutionary Game. The fisheries manager does not inherit his/her strategies but chooses them, and payoffs can take the form of monetary profits or other utility related metrics. While the fish can only adapt to the choices of the manager, the manager as a rational player can anticipate the ecological and evolutionary consequences of his/her decisions on nature, and therefore benefit from a lead position in the game.

The manager may want to anticipate the ecological but not the evolutionary consequences of his/her actions. Following Brown and Parman, we term this strategy “ecologically enlightened management” [22]. As we shall see, such a manager will drive the game to a Nash equilibrium. Subsequently, the fish will evolve to their ESS, in response to manager’s actions. Given this ESS, the manager cannot improve profits through a unilateral change in strategy.

Conversely, the manager may want to anticipate both ecological and evolutionary consequences. This manager engages in “evolutionarily enlightened management”, by optimizing profits knowing that the fish will evolve to their ESS [22, 28]. Actions of such a manager will lead to a Stackelberg equilibrium. At this equilibrium, the manager, with knowledge that the fish will evolve to their ESS in response to the manager’s strategy, can do no better.

Alternatively, the manager may use artificial selection or genetic modifications to dictate the evolutionary strategy of the fish. We will extend our analysis to this form of domestication. In getting to choose the fish’s strategy, the manager as leader in this game can achieve a team optimum. This results in the maximum achievable payoff to the manager because, in a sense, the fish are now “cooperating” with the manager.

This paper is organized as follows. In the next section we introduce a Stackelberg Evolutionary Game of fisheries management where the fish evolve their adult size and the manager can select harvesting rate and net size. We then introduce and analyze the three management strategies mentioned above (ecologically enlightened, evolutionarily enlightened and domestication) and the corresponding outcomes for the two players, first in the context of just varying harvesting rates, and then with varying net sizes as well. In particular, we highlight the benefits of an evolutionarily enlightened approach for both the fisheries manager and the fish. We conclude by drawing some general conclusions relating to fisheries management and by stressing the importance of developing Stackelberg Evolutionary Game theory for evaluating decisions in games involving evolving biological systems [29].

## 2 Materials and methods

### 2.1 Fisheries management as a game

We model fisheries management as a Stackelberg Evolutionary Game between a manager and a fish population (Table 1 provides a summary of strategies, parameters and functions). We extend the model of Brown and Parman [22] in four critical ways by 1) considering a background mortality rate on the fish, 2) permitting the manager to vary net size as well as fishing intensity, 3) considering the gains in yield produced from domesticating the fish under harvest, 4) considering a cost term for fishing.

**Table 1:** Variables, parameters and functions used in the game.

**Variables**	**Meaning**
*u* *H* *z*	Size of fish at maturity Instantaneous harvesting rate Net size
**Parameters**	**Meaning**
*R* *s* *d* *g* *c*	Intensity of density dependence Maximum fecundity rate per unit of adult size Background mortality rate Growth rate of juveniles Cost for fishing
**Functions**	**Meaning**
*P*(*H*, *u*, *z*) *M*(*u*)	Probability that juveniles survive harvesting till adulthood Probability that juveniles survive natural mortality till adulthood

We consider a fish species that begins as a neonate whose initial size approaches 0, and then grows at a linear rate *g* until an adult size *u*. As an adult, the fish switches from growth to producing neonates at a rate *su* proportional to its adult size. Both juvenile and adult fish suffer a background mortality rate *d*. Size at reproduction *u* is the fish’s evolutionary strategy. The manager’s strategy includes setting the harvesting rate *H* and the net size *z*. All fish greater than or equal to size *z* are subject to harvesting. For evaluating the eco-evolutionary dynamics of the fish, we define the expected per capita growth rate of a focal individual that reaches adulthood at size *v* in a population of fish that reaches adulthood at size *u*. For the ecology of the fish, we assume that the actual production of neonates declines with the adult population size of fish *N* [30]. By considering the focal fish’s strategy *v* and the strategy predominate in the fish population *u*, we have the following fitness generating function *G*:

$$\begin{array}{c} G\left(v,u\right)=svP\left(v\right)M\left(v\right)e^{\frac{-uN^{*}}{R}}-H^{\prime }-d, \end{array}$$ (1)

where *H*′ is the effective mortality rate from fishing: *H*′ = 0 if *z* > *u* and *H*′ = *H* if *z* ≤ *u*.

The *G*-function (1) gives the expected per capita growth rate of a focal fish of adult size *v* within a population of fish that matures at size *u* that has achieved its equilibrium adult population size of N*, and that is subjected to fishing rate *H* by nets of size *z*. The terms *P*(*v*) = *e*^−*H*(*v* − *z*)/*g*^ and *M*(*v*) = *e*^−*dv*/*g*^ give the probabilities of the focal fish’s neonates surviving harvesting and natural mortality, respectively, to reach adulthood. Note that the neonates suffer the size-independent natural mortality rate throughout their growth to adult size, whereas mortality from fishing only occurs when the juvenile fish reach the size of the net *z*. If net size is greater than or equal to *v*, then no juveniles spawned by the focal fish will be harvested: *P*(*v*) = 1 for *z* > *v*. Each adult fish’s potential to produce neonates declines with the total biomass of adult fish *uN* and increases with some pool of limiting resources *R*. The time it takes for a fish in the population to reach adulthood is $T=\frac{u}{g}$. This means that adult recruitment at time *t* results from neonates that were produced by the adult fish population at time *t* − *T*. Thus, the population dynamics of adult fish with strategy *u* at time *t* is given by:

$$\begin{array}{c} \frac{d\;N_{t}}{d\;t}=suP\left(u\right)M\left(u\right)N_{t-T}e^{\frac{-uN_{t-T}}{R}}-H^{\prime }N_{t}-d\;N_{t}, \end{array}$$ (2)

from which we obtain the expression for the equilibrium population size N*:

$$\begin{array}{c} N^{*}=\frac{R}{u}\log\frac{suP(u)M(u)}{H^{\prime }+d} \end{array}$$

### 2.2 How the fish see the game

Evolution by natural selection will favor the fish size that maximizes the expected fitness of a focal individual within the context of the sizes and densities of other fish. In our game, the evolutionarily stable strategy (ESS) is the adult fish size u* that cannot be invaded by any focal individual using a different strategy *v* ≠ u*. Thus *v* = u* maximizes *G* in a population using u* at N*. Applying the ESS maximum principle [27] to find the ESS value of u* gives:

$$\begin{array}{c} u^{*}\left(H,z\right)=\{\begin{array}{c} \frac{g}{H+d},\;\text{when}\;z\leq \frac{g}{H+d}\\ \frac{g}{d},\;\text{when}\;\frac{g}{d}<z\\ z,\;\text{when}\;\frac{g}{H+d}<z<\frac{g}{d}. \end{array} \end{array}$$ (3)

**Fig 1:**
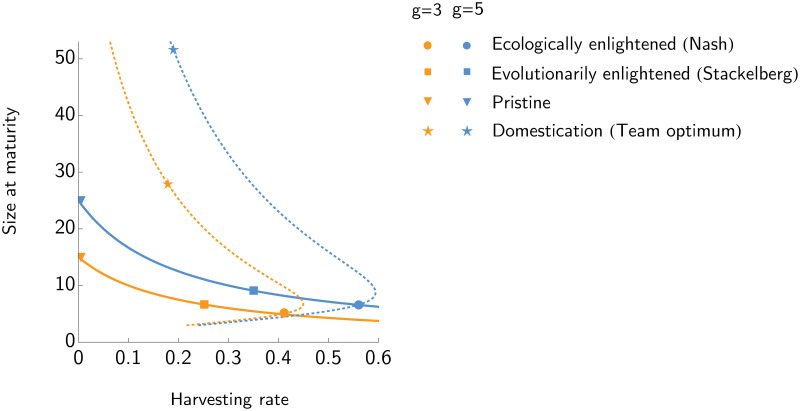
The best responses of the fish and of the manager. The ESS size of the fish (solid line) and the best response of the manager (dotted line) for two different values of the juvenile growth rate: *g* = 3 (orange) and *g* = 5 (blue). For this example, *R* = 4, *z* = 3, *d* = 0.2, *c* = 5 and *s* = 1. The ESS size of the fish increases with juvenile growth rate and decreases with harvesting rate, the optimal harvesting rate for the manager increases with juvenile growth rate. The Nash equilibrium lies at the intersection of the ESS curve of the fish and the best response curve of the manager, the Stackelberg equilibrium and the pristine lie on the ESS curve of the fish but not on the best response curve of the manager, while the team optimum lies on the best response curve of the manager, but not on the ESS curve of the fish.

The first condition in (3) ($u^{*}=\frac{g}{H+d}$) is found by taking the derivative of *G* with respect to *v*, then setting *v* = *u*, and finally setting this expression equal to zero and solving for u*. In a pristine environment, prior to any fishing, the adult size should increase with their growth rate and decline with the natural mortality rate: $u^{*}=\frac{g}{d}$. Fishing imposes an additional mortality rate on adults and so the ESS fish size will decline with the sum of harvesting and background mortality (Fig 1). The second condition on the ESS fish size in (3) comes into effect if harvesting becomes too intense or the net size too large. In the extreme, if $z>\frac{g}{d}$ then the effective harvesting rate is *H*′ = 0, no matter the actual attempted harvesting rate *H*. As no fish are harvested they retain their pristine size. But, what if the net size is smaller than the fish’s pristine size and the attempted harvesting rate is so intense as to create a situation where $\frac{g}{H+d}<z<\frac{g}{d}$? In this case, the ESS fish size becomes the net size: u* = *z*. The ESS of the fish can be represented as a best response curve in the (*H*, *u*)-space (Fig 1). The fish’s best response curve shows how the ESS size of the fish u*(*H*) decreases with the harvesting effort.

### 2.3 How the manager sees the game

We assume that the manager’s goal is to maximize a profit function *Q*, described as the difference between yield and cost. Yield function is defined as a rate of harvested biomass. For simplicity, we assume that the per biomass value of the catch is constant and independent of the actual size of the fish. Thus, a fish that is half the size of another has half the value. The yield comes from two sources. The first one is the harvesting of adult fish and the second one is the harvesting of juvenile fish that are larger than the net size. The first term is straightforward while the second is a rather involved integral, as the harvest of juveniles occurs from the time a juvenile is of size *z* (let this be time *τ*) and continues to time *T* when it reaches adulthood at size *u*. Both components are weighted by the size of the fish. Costs of fishing are represented by a term *cH*, where *c* is the per unit cost of fishing. Thus, the profit function can be written as:

$$\begin{array}{c} Q\left(N,u,H,z\right)=HuN^{*}+H\int_{\tau }^{T}gxe^{-H(x-\tau )}e^{-d(x-\tau )}\frac{\left(H+d\right)N^{*}}{P\left(u\right)e^{-d(T-\tau )}}dx-cH, \end{array}$$ (4)

where $\tau =\frac{z}{g}$ and $T=\frac{u}{g}$ are the limits of integration and $\frac{\left(H+d\right)N^{*}}{P\left(u\right)e^{-d(T-\tau )}}$ gives the number of juvenile fish alive at age $\frac{z}{g}$ required to keep an equilibrium population size of N*, given the harvest rate *H* and the adult size *u*. The integral in (4) can be solved explicitly and the profit becomes:

$$\begin{array}{c} Q\left(u,H,z\right)=\frac{HR}{u(H+d)}\log(\frac{sP(u)uM(u)}{H+d})\cdot (\frac{z(H+d)+g}{P\left(u\right)M\left(u\right)e^{\frac{du}{g}}}-g)-cH. \end{array}$$ (5)

### 2.4 The manager’s dilemma in setting the harvesting rate

We assume that the manager selects a harvesting rate and a net size in order to maximize profit. We will start with a manager that adjusts just the harvesting rate. We consider three approaches of the manager: 1) Ecologically enlightened (Nash), 2) Evolutionarily enlightened (Stackelberg), and 3) Domestication (team optimum).

#### 2.4.1 The ecologically enlightened manager

The ecologically enlightened manager recognizes the effects of harvesting on the population size of fish, but sees the adult size of the fish as fixed. This provides a tempting, short-term optimal strategy. The manager assumes that evolution is too slow to be of consequence, or that the fish cannot evolve. For determining the optimal harvesting rate, the manager considers the effect of *H* on N* and incorporates this into the profit function. The optimal harvesting rate *H*_*ECO*_ comes from maximizing profit *Q* with respect to *H*, while holding *u* constant: *H*_*ECO*_ = argmax_*H*_ *Q*(*u*, *H*, *z*) (see S1 Appendix for the analysis of the equilibria and their stability properties). In this way, H* becomes a function of *u*, and the curve of H* with respect to *u* is the best response curve of the ecologically enlightened manager in response to the size of the fish (Fig 1).

There are three possible outcomes for this style of management. First, there is the optimal harvesting rate when the fish are of the pristine size *u*_*PRIS*_. The manager is tempted to adopt the harvesting rate which maximizes the profit assuming that the fish keep their pristine size *u*_*PRIS*_. If the fish do not evolve, then this state can persist indefinitely. However, if the fish evolve, the only strategy that preserves their pristine size is *H*_*PRIS*_ = 0 (Fig 1, triangle marker). Second, the fish size begins to evolve towards $\frac{g}{H_{PRIS}+d}$. This creates a transient dynamic, where the fish and/or the manager alter their strategies, the fish changing in the direction of $\frac{g}{H+d}$ and the manager shifting along his/her best response curve as the fish evolve to a smaller size. Third, if the fish and manager have sufficient time, an eco-evolutionary equilibrium happens where the best response curve of the fish intersects with that of the manager (Fig 1, circle marker). This is a Nash equilibrium that combines elements of evolutionary game theory and classical game theory. The fish are at an ESS: $u_{ECO}=\frac{g}{H_{ECO}+d}$. At this point the fish are evolutionarily stable in that no individual can increase its fitness by unilaterally changing its size, and the fish are ecologically stable in that their expected per capita growth rate is 0 at N*. For the manager this is a no regret strategy: given the size of the fish, the manager has no incentive to change the harvesting rate.

When the net size is *z* = 0, the intersection of best response curves is always the ESS for the fish and the Nash for the manager. Not necessarily so for a larger net size. If excessive harvesting reduces the fish size below the net size z (this actually occurs at $z=\sqrt{\frac{ge^{\left(1+c/R\right)}}{s}}$, see S1 Appendix), the harvesting effort has to be modulated down ($H_{ECO}=\frac{g}{z}-d$) in order to keep the fish from evolving to be just below *z* and slip through the net. An interesting eco-evolutionary cycle could happen when $H_{ECO}+d>\frac{g}{z}$ for *u* = *z*: fishing could drive the size of the fish below *z*, which would then make fishing cease (unless the manager reduces *z*) until fish evolved to be larger again.

#### 2.4.2 The evolutionarily enlightened manager

The evolutionarily enlightened manager anticipates that the fish will evolve in response to harvesting. Thus, he/she incorporates both the ecological and the evolutionary consequences of harvesting into the profit function *Q* and selects the *H*_*EVO*_ that maximizes profit: *H*_*EVO*_ = argmax_*H*_ *Q*(u*, *H*, *z*). This will not be a point on the manager’s best response curve, but a point on the fish’s ESS curve where profit is maximized (Fig 1, square marker). So long as the net size is not too large, the *H*_*EVO*_ will always be less than *H*_*ECO*_. The manager will sacrifice short-term opportunities and maintain higher profits in the long-term by preserving both the ecological and evolutionary states of the fishery. If the net size is sufficiently large then the *H*_*ECO*_ coincides with the *H*_*EVO*_, but this occurs under the unlikely circumstances that the harvesting rate is reduced to keep the fish from evolving below the net size (see S1 Appendix for the analysis of the equilibria and their stability properties).

The game evolves towards a new eco-evolutionary equilibrium, which is different from the one reached by the ecologically enlightened manager. The fish evolve to their ESS size of $u_{EVO}=\frac{g}{\left(H_{EVO}+d\right)}$ and a stable equilibrium population size of fish occurs at $N_{EVO}^{*}$. The manager’s strategy no longer leads to a Nash equilibrium, as he/she could benefit (at least temporarily) by unilaterally changing the harvesting rate. But, it is a Stackelberg strategy where the manager anticipates the strategy of the other players. Thus, this manager’s strategy will always be as good or better than the manager’s Nash solution.

#### 2.4.3 Domestication

We consider a manager that can “domesticate” the fish by selecting the desired fish size using artificial selection or genetic modification. We imagine that the fish are still free-range in the sense of being stocked in the sea or lake. Many impracticalities come to mind, and fish farming may be more likely altering many of the model’s parameters rather than just fish size and harvesting rates. Regardless, our example of domestication shows the temptation to make the fish size *u* a control variable of the manager (along with *H*), and it illustrates another game theory solution known as the team optimum. In terms of maximizing profits, the manager now has a vector valued strategy of maximizing with respect to *H* and *u*: (*H*_*DOM*_, *u*_*DOM*_) = argmax_(*H*, *u*)_ *Q*(*u*, *H*, *z*). The team optimum lies on the best response curve of the manager but outside ESS curve of the fish (see Fig 1, star marker).

### 2.5 The manager’s dilemma in setting the net size

The manager can also choose the net size. It is likely that changing *H* can occur more frequently and nimbly than changing the net size. Net size represents a substantial investment in equipment that is meant to last many years. Whereas *H* can change seasonally, net size likely changes over spans of years. There is one exception when “net size” represents the safe release of individuals above or below a certain size. For this reason, we assume that the manager can change both harvesting rate and net size with immediate effect and without additional costs.

The ecologically enlightened manager will set both *H* and *z* without considering how the fish will evolve, resulting in the Nash solution. The evolutionarily enlightened manager aiming for a Stackelberg solution can set both *H* and *z* with the anticipation that the fish will evolve to size $\frac{g}{H_{EVO}+d}$. Domestication permits the manager to select *H*, *u* and *z* to reach the team optimum of the game. For the ecologically and evolutionarily enlightened managers, selecting net size only indirectly influences adult fish size, when harvesting is not so intense as to drive the fish’s size to at or below *z*. In this case, net size per se does not select for adult fish size. However, the selection of net size will influence *H*_*ECO*_ and *H*_*EVO*_, and thus fish size.

## 3 Results

### 3.1 Game with the manager having a one-dimensional decision space

**Fig 2:**
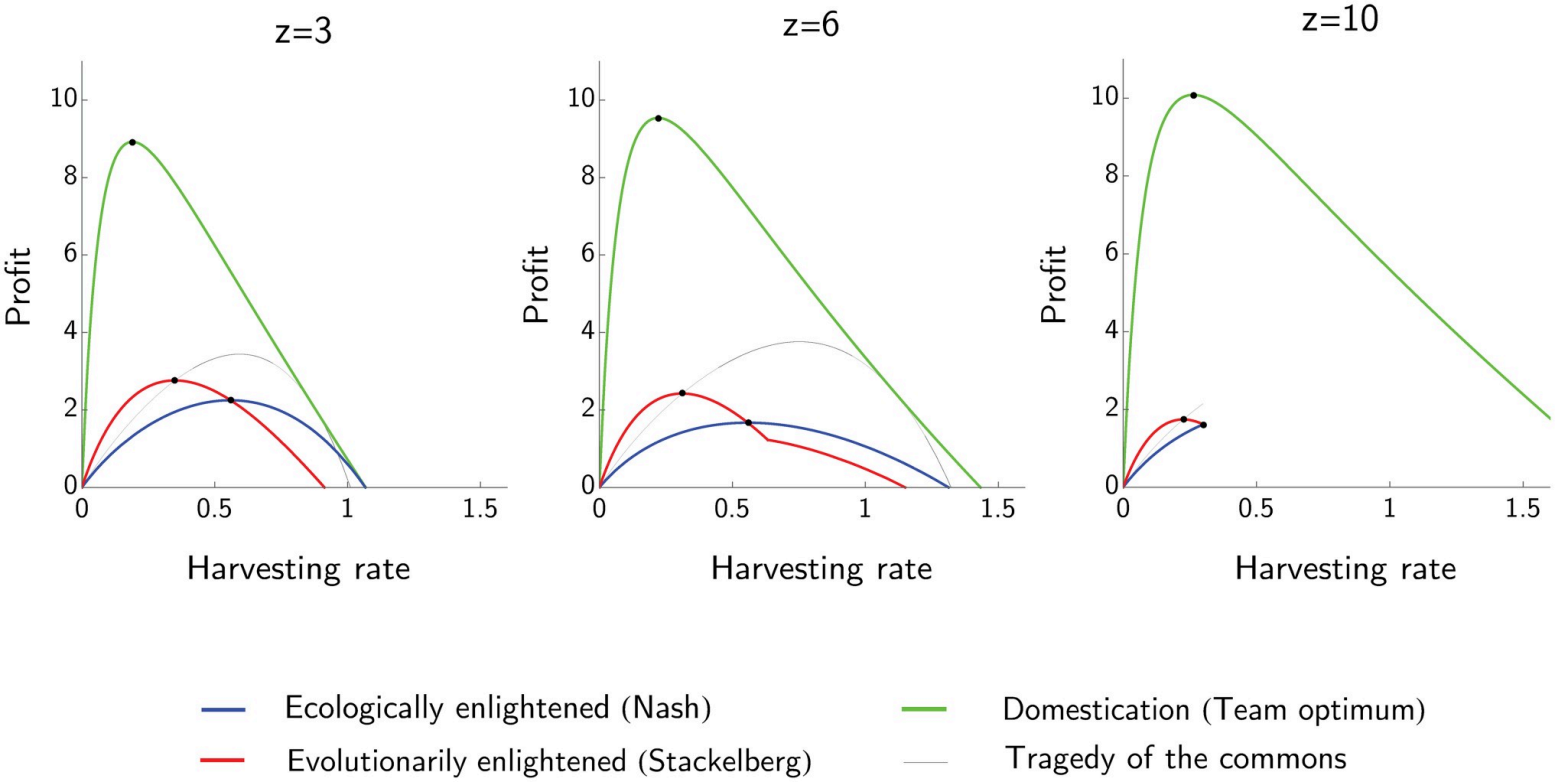
The effect of harvesting rate on the profit for three management strategies: Ecologically enlightened, evolutionarily enlightened and domestication. Each panel corresponds to increasing net size: *z* = 3, *z* = 6 and *z* = 10. In all three cases: *R* = 4, *s* = 1, *d* = 0.2, *g* = 5 and *c* = 5. The ecologically enlightened manager considers the size of fish at maturation as fixed (*u*_*ECO*_) and selects the harvesting rate *H*_*ECO*_ that maximizes the profit with this in mind. The evolutionarily enlightened manager assumes that the size of fish at maturation is the ESS u* and selects a harvesting rate *H*_*EVO*_ that maximizes the profit accordingly. The profit curve for the evolutionarily enlightened management intersects the profit curve for the ecologically enlightened management at its maximum. The evolutionarily enlightened approach leads to higher profits with a lower harvesting rate than the ecologically enlightened one, but is susceptible to the tragedy of the commons. The curve “Tragedy of the commons” shows how the profit varies with *H* when the size of the fish is fixed at *u*_*EVO*_. The profit curve for domestication lies above all the others.

The effect of harvesting effort *H* on the profit for the three management strategies is illustrated in Fig 2. The profit function of the evolutionarily enlightened (Stackelberg) manager has a higher global maximum than the profit function of the ecologically enlightened (Nash) manager. Moreover, this Stackelberg maximum is reached at a lower harvesting rate than the Nash one. As it must, the evolutionarily enlightened profit curve intersects the ecologically enlightened one from above and at its peak. This means that the maximum profit of the evolutionarily enlightened manager is unavailable to the ecologically enlightened one, but the maximum profit of the ecologically enlightened manager is available to the evolutionarily enlightened one. In other words, an evolutionarily enlightened (Stackelberg) manager can always reach the Nash solution, but not vice versa. As happens in classical Stackelberg games, the Stackelberg outcome is for the manager as good or better than the Nash outcome.

The ecologically enlightened strategy is stable in the ecological time, there is no benefit to changing harvesting strategies until the fish themselves evolve. On the contrary, the evolutionarily enlightened strategy invites a tragedy of the commons. The evolutionarily enlightened manager could achieve a higher profit in the short-term by increasing harvesting to *H*_*TRAG*_ > *H*_*EVO*_, while the fish have size *u*_*EVO*_ (Fig 2). This holds only in ecological time. In evolutionary time, fish will decrease in size and the profit will consequently drop. We can say that both (*H*_*ECO*_, *u*_*ECO*_) and (*H*_*EVO*_, *u*_*EVO*_) are stable in evolutionary time while (*H*_*TRAG*_, *u*_*EVO*_) is not. The third management strategy is domestication, which corresponds to the game-theoretic concept of team optimum. In this case fish can be selected to be well over the threshold $u=\frac{g}{d}$, and the profit for the manager is much higher than the one obtained by exploiting wild stocks. Thus, regardless of the value of *z*, the following inequalities hold:

$$\begin{array}{c} H_{EVO}\leq H_{ECO},\\ u_{ECO}\leq u_{EVO}\leq u_{DOM},\\ Q\left(u_{ECO},H_{ECO},z\right)<Q\left(u_{EVO},H_{EVO},z\right)<Q\left(u_{DOM},H_{DOM},z\right). \end{array}$$

Note that we consider the population *N* to be at the equilibrium.

### 3.2 Game with the manager having a two-dimensional decision space

**Fig 3:**
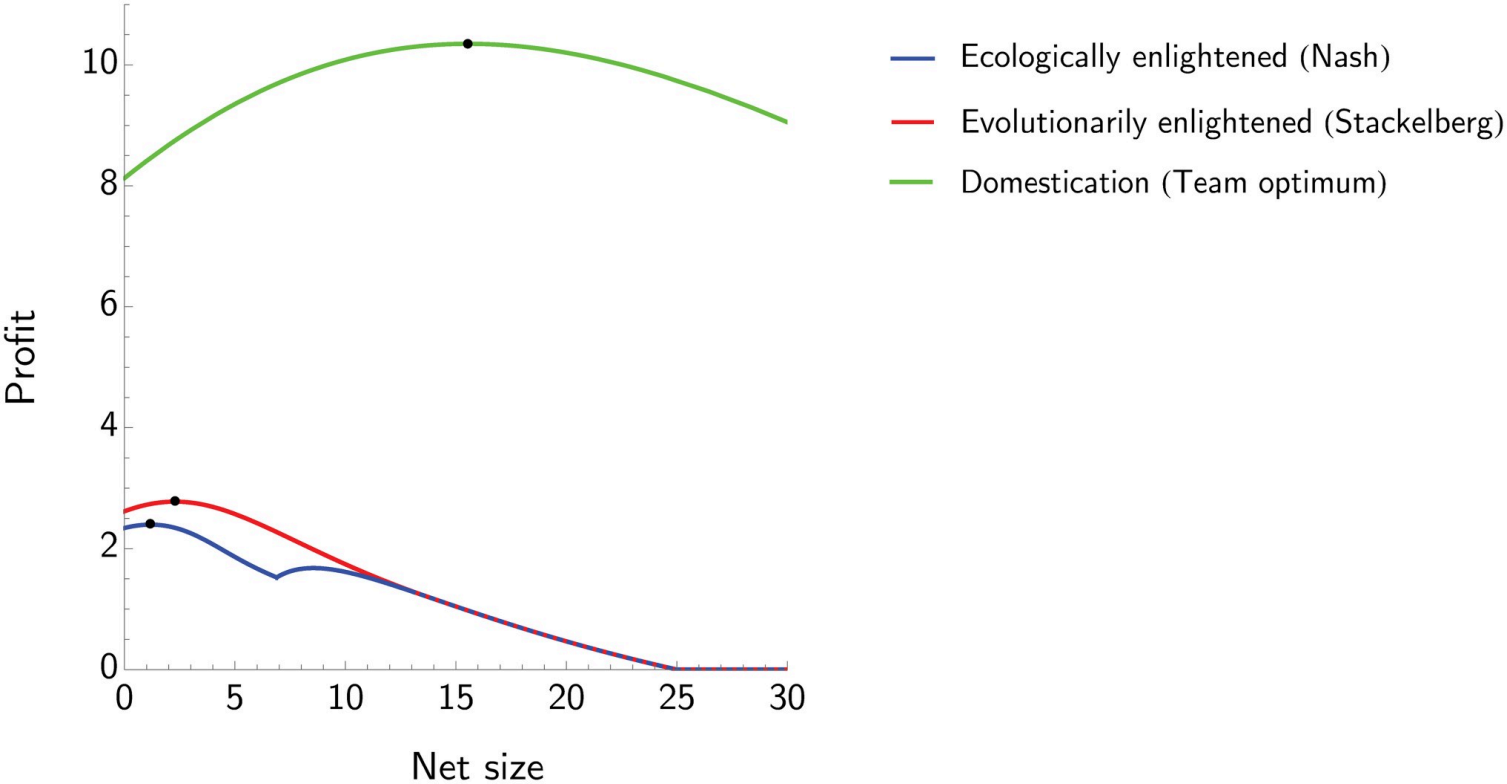
The effect of net size on the profit for three management strategies: Ecologically enlightened, evolutionarily enlightened and domestication. In this example *R* = 4, *s* = 1, *d* = 0.2, *g* = 5 and *c* = 5. The evolutionarily enlightened (Stackelberg) curve reaches a higher peak at a higher net size than the ecologically enlightened (Nash) curve. The evolutionarily enlightened profit curve intersects the ecologically enlightened one from above and they coincide from that point onwards. Domestication leads to substantially higher profits and a large optimal net size.

The effects of net size *z* and harvesting rate *H* on profits for the three management strategies is showed in Fig 3. In particular, for each fixed *z* we maximize profit with respect to *H*, and then select the *z* corresponding to the highest profit.

For the ecologically enlightened manager, we have a local maximum in the region where $z\geq \sqrt{\frac{ge^{\left(1+c/R\right)}}{s}}$ and a global one for $z<\sqrt{\frac{ge^{\left(1+c/R\right)}}{s}}$. The unique optimal *z* for the evolutionarily enlightened manager lies in the region where $z<\sqrt{\frac{ge^{\left(1+c/R\right)}}{s}}$ as well. The profit to the evolutionarily enlightened manager at (*H*_*EVO*_, *z*_*EVO*_) is greater than that of the ecologically enlightened manager at (*H*_*ECO*_, *z*_*ECO*_), and is obtained by selecting a lower harvesting effort but a higher net size. Interestingly, if the net size is set above the threshold $z=\sqrt{\frac{ge^{\left(1+c/R\right)}}{s}}$, the interests of the ecologically enlightened manager and those of the evolutionarily enlightened manager differ. In particular, the first one aims at increasing *z* in order to reach the (local) maximum profit, while the evolutionarily enlightened manager has no incentive in further increasing *z* as his/her profit will decrease with *z*. Domestication corresponds again to the highest profit possible. Assuming that the population *N* is at the equilibrium, the following inequalities hold:

$$\begin{array}{c} z_{ECO}\leq z_{EVO}\leq z_{DOM}\\ H_{DOM}\leq H_{EVO}\leq H_{ECO}\\ u_{ECO}\leq u_{EVO}\leq u_{DOM}\\ Q\left(u_{ECO},H_{ECO},z_{ECO}\right)\leq Q\left(u_{EVO},H_{EVO},z_{EVO}\right)\leq Q\left(u_{DOM},H_{DOM},z_{DOM}\right). \end{array}$$

### 3.3 Sensitivity analysis

**Fig 4:**
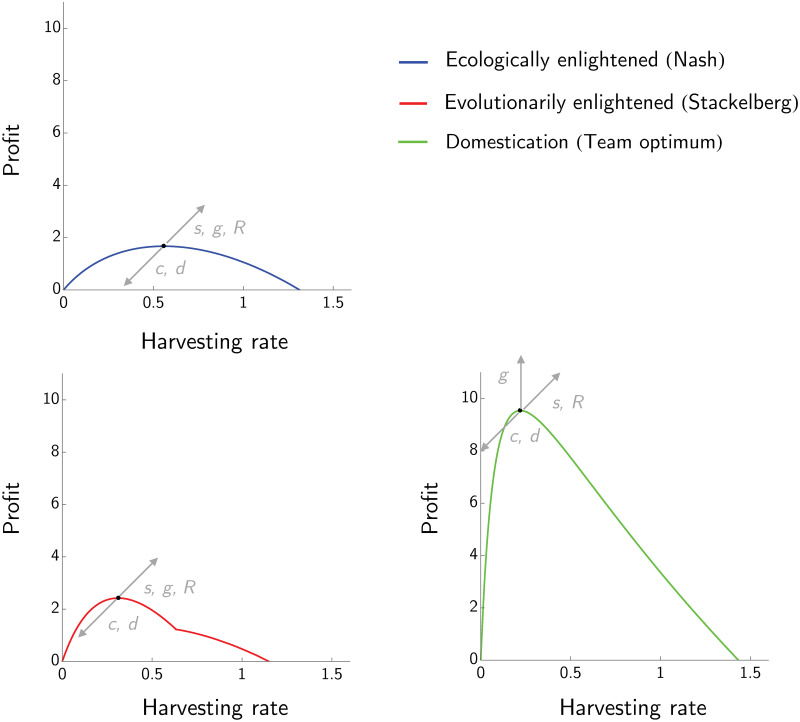
Sensitivity analysis of the three equilibria for the case where the manager can set only the harvesting rate. Sensitivity analysis showing how profit and harvesting rates change with the intensity of density dependence *R*, the maximum fecundity rate *s*, the natural mortality rate *d*, the growth rate of juveniles *g* and the cost of fishing *c* (*z* = 6). Arrows pointing up (down) indicate that the profit is increasing (decreasing) when the corresponding parameter increases, arrows pointing to the right (left) indicate that the harvesting rate at the equilibrium is increasing (decreasing) when the corresponding parameter increases. The profit at the ecologically enlightened and evolutionarily enlightened equilibria are directly proportional to *s*, *R* and *g* and inversely proportional to *c* and *d*. The harvesting rate at both equilibria increases when *s*, *g* or *R* increase and decreases when *c* or *d* increase. The profit at domestication equilibrium increases with *s*, *R* and *g* and decreases with *c* and *d*. The corresponding harvesting rate increases with *R* and *s* and decreases with *c*.

**Fig 5:**
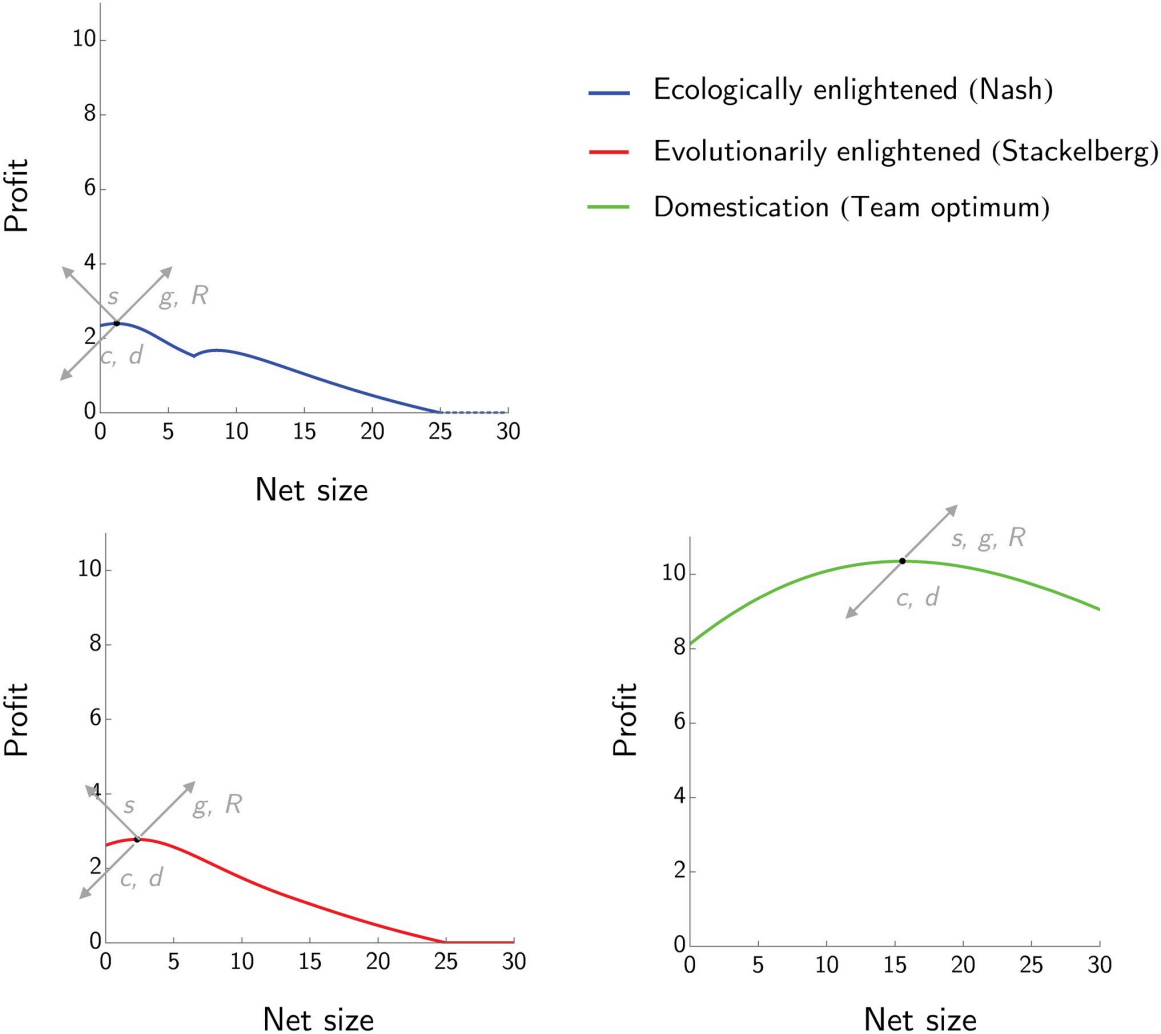
Sensitivity analysis of the three equilibria for the case where the manager can set both the harvesting rate and the net size. Sensitivity analysis showing how how profit and net sizes change with respect to the intensity of density dependence *R*, the maximum fecundity rate *s*, the natural mortality rate *d*, the growth rate of juveniles *g* and the cost of fishing *c*. The profit of the ecologically enlightened (Nash) manager and of the evolutionarily enlightened (Stackelberg) manager are directly proportional to *s*, *R* and *g* and inversely proportional to *c* and *d*. The optimal net size of both managers increases when *g* or *R* increase and decreases when *s* increases. Moreover, the optimal net size of both managers decreases with *c* and *d*. The profit from domestication and the corresponding optimal net size increase with *s*, *R* and *g* and decrease with *c* and *d*.

Given the three equilibria (Nash, Stackelberg and team optimum), we analyze how sensitive they and the corresponding profits are with respect to small variations of model parameters *s*, *R*, *g*, *d* and *c*. Fig 4 shows the sensitivity analysis for the case where the manager can only vary *H*, while Fig 5 shows the sensitivity analysis when manager adjusts both *H* and *z*. In the case where the manager chooses only the harvesting rate *H*, increasing the value of *s*, *g* or *R* will increase both the profit at the three equilibria and the corresponding optimal *H*’s, while increasing the value of *c* or *d* will decrease the maximum profit and the corresponding optimal *H*’s.

Similarly, in the case where the manager chooses both the harvesting rate and the net size, increasing the parameters *g* and *R* will increase profits for all three management strategies as well as the corresponding optimal *z*’s. Increasing *s* will increase the profit at all the equilibria and the corresponding optimal *z* in the case of domestication, but will decrease the optimal *z* in the case of ecologically or evolutionarily enlightened management. Increasing the juvenile growth rate *g* will increase both the optimal net size and the profits at all of the equilibria. As expected, increasing the natural death rate *d* will decrease maximum profits, and also the corresponding optimal net size *z*. Similarly, increasing the cost of fishing *c* will decrease profits and the corresponding optimal net size *z*. The sensitivity analysis shows that the effect of the parameters on profits does not depend on whether we consider *z* as a fixed or changing variable.

## 4 Discussion

Over-exploitation threatens the ecological sustainability of many of the world’s fisheries [2]. Ecological models aim to understand the incentives and consequences of overfishing, and suggest ecologically enlightened management strategies [31, 32]. This may not be enough. Intense or size-selective harvesting imposes selection pressure on important life-history traits. One of the most evident effects of fisheries-induced evolution, is the reduction of average body size at maturation [1, 33]. Such evolutionary effects can diminish stock biomass and sustainable yield in just a few generations, with far-reaching ecological and economic consequences [1]. This invites evolutionarily enlightened management strategies to promote both ecological and evolutionary sustainability [34, 35].

A large body of literature, including theoretical and experimental studies, show how fishing pressures drive many of the life history changes observed in fish stocks [11, 12, 20, 33, 36–38]. The highest rates of evolution have been associated with the most heavily exploited stocks [1, 39, 40]. In light of the ecological challenges of managing fisheries, it is tempting to dismiss the evolutionary changes as occurring too slowly or attribute changes to other environmental drivers [36, 41–43]. Dealing with evolutionary effects may not be urgent. An ecologically enlightened approach may be sufficient, but most likely not [42, 44].

The fisheries induced evolution can seriously impact the future viability of stocks [40, 42] by reducing yields and magnifying the consequence of over-fishing. Even small evolutionary changes can lead to the collapse of a heavily overexploited stock [23]. Once it has happened, the fisheries induced evolutionary changes may be slow to reverse or even irreversible [12, 45–47]. Fisheries managers should consider rapid evolutionary changes as possible and likely [48]. Fisheries are in need of theory, empiricism and the application of evolutionarily enlightened management [1, 8, 28, 40, 49, 50].

In this work, we formalize the fisheries management problem as a Stackelberg evolutionary game, a novel framework for including the manager as a rational player and the fish as evolving players. In this Stackelberg (or leader-follower) game, the fisheries manager is the leader capable of anticipating the consequences of his or her actions on the ecological (fish population size) and evolutionary (fish size at maturation) dynamics of the fish. Whether in the presence or absence of harvesting, the eco-evolutionary dynamics of the fish converge on an ESS in accord with the principles of evolutionary game. While most models consider harvesting intensity, net size is also an important control variable of the manager [12, 40]. Setting minimum catch size limits provides an important complementary management tool to decrease the evolutionary impact of fishing and deserves attention [40, 51]. While many studies have focused on the effect of the net size on evolution, ignoring optimal strategies, or on the optimal net size, ignoring evolution [52], our game-theoretic approach allows to consider both the optimal strategies of the fisheries manager and the evolution of the fish stock. In addition to considering both harvesting effort and net size, our modelling is expansive in terms of management contexts that can include the original pristine environment (no harvesting), ecologically enlightened management, evolutionarily enlightened management, and domestication. Within these contexts we can consider both transient dynamics and the equilibrium states.

To achieve our goal of comparing management strategies, we extended the model of Brown and Parman [22] to include 1) a background mortality rate, 2) a cost to fishing, and 3) the additional strategy of choosing the net size. These adjustments add realism and scope of application. Thus, we could analyze the game for cases where the manager can only control the harvesting effort and for cases where the manager sets both the harvesting effort and the net size. In actual fisheries, the manager can change harvesting effort both within and between fishing seasons whereas changing net size happens less often because fishing gear is expected to last multiple years and seasons.

In our Stackelberg evolutionary game, the strategy of the fisheries manager in terms of the harvesting rate and net size directly influences the fish equilibrium population size and ESS maturation size. This is the first step of an evolutionary impact assessment (EvoIAs) advocated by the promoters of evolutionarily enlightened approaches [1, 49]. In our model, when the harvesting rate or the net size are low, the ESS maturation size equals the ratio between the juvenile growth rate and the combined death rate from fishing and natural mortality. Increasing the harvesting rate selects for a decreased ESS size at maturation, independent of the net size. Conversely, when the harvesting rate or the net size are high, the ESS maturation size equals the net size, independent of the harvesting rate, but increasing the net size leads to an increased size at maturation. Our predictions are consistent with those of Andersen and Brander [53] and contrary to Faig [25], who suggested that increasing the net size would decrease the size at maturation. As proposed by [49], the second step of an evolutionary impact assessment includes analyzing how the evolutionary changes that take place in the exploited fish stocks may alter their utility to stakeholders or society [1, 54]. To this aim, we compared different possible outcomes of the game, dependent on what information about the fish eco-evolutionary dynamics the fisheries manager knows or chooses to take into account.

Our Stackelberg evolutionary game theory approach permits us to jointly analyze the eco-evolutionary dynamics of the fish and their economic consequences to the stakeholders under four management strategies. The management approaches and their game theoretic equivalents are summarized in Table 2.

**Table 2:** The possible approaches of the manager, the corresponding game-theoretic concepts and the strategies of the manager and the fish.

Manager’s approach	Corresponding game-theoretic concept	Strategies
Ecologically-enlightened	Nash	$H_{ECO}=\text{arg}\max_{H}Q\left(N^{*}\left(u_{ECO},H\right),u_{ECO},H\right)$ $u_{ECO}=\arg\max_{u}G\left(N^{*}\left(u,H_{ECO}\right),u,H_{ECO}\right)$
Evolutionarily-enlightened	Stackelberg	$H_{EVO}=\text{arg}\max_{H}Q\left(N^{*}\left(\gamma_{u}\left(H\right),H\right),\gamma_{u}\left(H\right),H\right)$ *u*_*EVO*_ = *γ*_*u*_(*H*_*EVO*_) with $\gamma_{u}\left(\tilde{H}\right)=\text{arg}\max_{u}G\left(N^{*}\left(u,\tilde{H}\right),u,\tilde{H}\right)$
Domestication	Team optimum	$\left(H_{DOM},u_{DOM}\right)=\text{arg}\max_{\left(H,u\right)}Q\left(N^{*}\left(u,H\right),u,H\right)$
Pristine	-	*H_PRIS_* = 0 $u_{PRIS}=\arg\max_{u}G\left(N^{*}\left(u,0\right),u,0\right)$

In each of these situations, there is a single optimal harvesting rate that maximizes the fisheries manager’s profit function for a given net size. The Pristine case imagines an unexploited fishery and has the manager choosing an optimal harvesting rate and net size under the assumption that fisheries-induced evolution will not or cannot happen. Under an ecologically enlightened strategy, evolution does happen, but the manager only anticipates the ecological and not the evolutionary consequences of harvesting intensity and net size. This leads to a Nash equilibrium of the Stackelberg evolutionary game. Under an evolutionarily enlightened strategy, the manager anticipates the ESS of the fish when selecting the optimal harvesting intensity and net size. The outcome leads to a Stackelberg equilibrium. From classical Stackelberg game theory, it is well known that the leader’s payoff from the Stackelberg equilibrium is at least as good, and generally much better than the leader’s payoff at the Nash outcome. This result still holds for Stackelberg evolutionary games both with one or two decision variables for the leader, as shown by the profit curves of the manager (Figs 2 and 3): it is a better strategy for the fisheries manager to anticipate and steer both the ecological and evolutionary responses of the fish. Compared to the ecologically enlightened strategy, the evolutionarily enlightened strategy leads to higher profits, lower harvesting rate, the same or larger net size, a higher standing crop of fish, and larger sized fish at maturation.

The Stackelberg solution could be further improved if the leader could select harvesting intensity, net size and the fish size at maturation. Under this domestication strategy, fish body size would no longer be determined by natural selection. For if it were, the fish would evolve to a smaller body size. The manager would achieve the desired size through genetic engineering or artificial selection. These artificially selected fish could then be released into the marine system or farmed. However, if the released fish can go feral, continuous replenishment of the domesticated strategy would be required to keep the fish from evolving to a smaller body size. Whereas ecologically enlightened and evolutionarily enlightened management result in smaller fish than originally occurring under pristine conditions, the profit maximizing domestication strategy breeds fish that are larger than pristine size. While this might be seen as an idealized case which is not applicable to the case of harvesting wild stocks, this solution concept is not so far-fetched and could fit the case of fish farming or fish stocking, which is widely practiced and involves release into the wild. In principle and in practice, the hatchery can both select on the adult fish and rear the young for release. In this sense, one can select for larger size, delayed reproduction and other desirable characteristics without having the fish be self sustaining in the wild, as new individuals are constantly restocked from the hatchery. From a game-theoretic point of view, domestication corresponds to having cooperative followers, who help the leader to obtain the maximum possible payoff, disregarding their own benefits (team optimum). With domestication, the fish and manager’s strategies correspond to an optimal inverse Stackelberg strategy [55, 56]. In terms of utility to the stakeholders and ESS fish size at maturation: Domestication > Pristine > Stackelberg > Nash. While favorable if the fish do not evolve, the pristine size is just a transient and not sustainable under fishery-induced evolution. Our results suggest that ignoring evolution provides short-term rewards (current payoff), but turns out to be myopic in the long run. With the Stackelberg (evolutionarily enlightened) approach the ESS fish size is substantially larger than with the Nash (ecologically enlightened) approach for low values of the net size, and at least as large for high values of the net size. Generally, the larger net size of the Stackelberg solution compared to the Nash solution can further accentuate the difference in ESS fish sizes between the two management strategies. In this way, the Stackelberg solution can maintain an ESS fish size closer to the original pristine size and provide a higher return to the stakeholders. This result is in line with recent studies confirming that maximizing the sustainable profit and mitigating negative effects of fishery-induced evolution are not necessarily in conflict [51]. The Stackelberg (evolutionarily enlightened) solution is feasible (unlike pristine), can be applied to wild stocks (unlike domestication/team optimum) and, most importantly, can better align harvesting and conservation interests. Eikeset et al. compared two scenarios for the management of an evolving fish stock that, like our approach, depended on whether evolution is accounted for or ignored. Unlike our results, they concluded that the optimal harvesting rate for the two cases is almost identical [23].

Our model only investigated the role of a minimum size limit, which is common in many fisheries. However, the optimal size at which to harvest fish may fall into a range where the smallest and largest fish escape harvesting [57]. There is evidence that preserving large individuals can mitigate fisheries-induced evolution making fish stocks more profitable [35, 42, 51, 57, 58]. For future consideration our model could include a harvesting regime that only takes individuals above and below a certain size. Also, our model could be extended to include indeterminate growth even after maturation, adding realism for certain fish species.

The benefits from taking an evolutionarily enlightened approach accrues from moderating harvesting rates and increasing net size. However, the actual benefit depends on the time horizon considered by the manager and the time required for the new ESS to take place. In this paper we assumed the evolutionary speed to be fixed, but in reality we do not know how fast evolution takes place. Our assessments of the different management strategies were based on equilibrial states. While we have shown that these eco-evolutionary equilibria are stable (see S1 Text), we did not analyze the transient dynamics towards the ESSs of the fish. Depending on the evolutionary speed, we might or might not reach the equilibria mentioned in this work. Empirical data and experimental studies [1] could suggest the speed at which population and evolutionary dynamics occur. For this reason, future research will focus on fitting the model on such data, in order to determine how realistic it is that we reach these equilibria in reasonable time. From a game-theoretic viewpoint, the optimal strategies summarized in Table 2 are derived assuming that the game has perfect information. However, in real life many factors are unknown, like the just-mentioned speed of evolution or the relative importance of other external evolutionary forces, which could influence the evolutionary trajectories and ecological variability of harvested fish stock. For this reason, we plan to relax the hypothesis of perfect information in future work, when considering the dynamics of the system and not only the equilibria.

In this paper, we investigate a Stackelberg Evolutionary Game between a single fisheries manager as a leader and monomorphic fish population as followers. In our future research we wish to expand this game into situations with multiple leaders and heterogenous followers. For this purpose, experiments in behavioral economics, which investigate how individuals deviate from predicted behavior when exploiting common-pool resources, will be used to validate our model parameters, to obtain realistic predictions [59–63].

Overall, the management suggestions that emerge from conceptualizing fisheries management as a Stackelberg evolutionary game align well with others’ work and suggestions. Reduced harvesting rates are known to allow fish stocks to rebuild and support higher yields. Moreover, altering size-selectivity through regulation aimed at enlarging the net size has already been recognized as a complementary tool to reduce fisheries-induced evolution [49, 54]. On a broader level, we advocate for the use of Stackelberg evolutionary game theory as a tool for providing insights into the management of evolving resources, pests and diseases [29]. Recommending that harvesting rates be curtailed to manage the fish’s ESS has direct parallels in scaling back the use of antibiotics to prevent resistant bacteria, of pesticides to reduce insect resistance, and of chemotherapies to reduce drug resistance in cancer [64, 65].

## Supporting information

S1 Appendix Concavity and stability analysis of the equilibria. (PDF)

## References

[1] Jørgensen C, Enberg K, Dunlop ES, Arlinghaus R, Boukal DS, Brander K, et al Ecology: managing evolving fish stocks. Science. 2007; 318: 1247–1248. 10.1126/science.1148089 18033868

[2] Hutchings JA, Reynolds JD. Marine fish population collapses: consequences for recovery and extinction risk. BioScience. 2004; 54(4): 297–309. 10.1641/0006-3568(2004)054[0297:MFPCCF]2.0.CO;2

[3] Dulvy NK, Sadovy Y, Reynolds JD. Extinction vulnerability in marine populations. Fish and fisheries. 2003; 4(1): 25–64. 10.1046/j.1467-2979.2003.00105.x

[4] Myers RA, Worm B. Rapid worldwide depletion of predatory fish communities. Nature. 2003; 423(6937): 280–283. 10.1038/nature01610 12748640

[5] Dieckmann U, and Heino M. Fishing drives rapid evolution. Swedish Research for Sustainability. 2004; 3(4): 18–19.

[6] van Wijk SJ, Taylor MI, Creer S, Dreyer C, Rodrigues FM, Ramnarine IW, et al Experimental harvesting of fish populations drives genetically based shifts in body size and maturation. Frontiers in Ecology and the Environment. 2013; 11(4): 181–187. 10.1890/120229

[7] Heino M, Diaz Pauli B, Dieckmann U. Fisheries-induced evolution. Annual review of ecology, evolution, and systematics. 2015; 46: 461–480. 10.1146/annurev-ecolsys-112414-054339

[8] Kuparinen A, Merilä J. Detecting and managing fisheries-induced evolution. Trends in Ecology & Evolution. 2007; 22(12): 652–659. 10.1016/j.tree.2007.08.011 17981361

[9] Hutchings JA, Fraser DJ. The nature of fisheries- and farming-induced evolution. Molecular ecology. 2008; 17(1), 294–313. 10.1111/j.1365-294X.2007.03485.x 17784924

[10] Heino M, Dieckmann U, Godø OR. Reaction norm analysis of fisheries-induced adaptive change and the case of the Northeast Arctic cod. ICES; 2002.

[11] Law R, Grey DR. Evolution of yields from populations with age-specific cropping. Evolutionary Ecology. 1989; 3(4): 343–359. 10.1007/BF02285264

[12] Dunlop ES, Enberg K, Jørgensen C, Heino M. Toward Darwinian fisheries management. Evolutionary Applications. 2009; 2(3): 245–259. 10.1111/j.1752-4571.2009.00087.x 25567878 PMC3352496

[13] Belgrano A, Fowler CW. How fisheries affect evolution. Science. 2013; 342(6163): 1176–1177. 10.1126/science.1245490 24311669

[14] Davis GRC. Magna Carta, Revised Edition. British Library 1989.

[15] FAO. *Code of Conduct for Responsible Fisheries*. FAO, Rome, Italy 1995.

[16] Nielsen M, Hoff A, Nielsen R, Andersen P. Structural Adjustment and Regulation of Nordic Fisheries until 2025. Nordic Council of Ministers; 2018.

[17] NOAA (National Oceanic and Atmospheric Administration). Atlantic highly migratory species; Atlantic Bluefin Tuna fisheries. Federal Register. 2005; 70: 159.

[18] Acheson JM, Knight J. Distribution fights, coordination games, and lobster management. Biological Conservation. 2000; 42(1): 209–238.

[19] Acheson J, Gardner R. Fishing failure and success in the Gulf of Maine: lobster and groundfish management. Maritime Studies. 2014; 13(1): 8 10.1186/2212-9790-13-8

[20] Heino M. Management of evolving fish stocks. Canadian Journal of Fisheries and Aquatic Sciences. 1998; 55(8): 1971–1982.

[21] Blythe S, Stokes T. Some consequences of size-selective harvesting on fitness and on yield. Mathematical Medicine and Biology: A Journal of the IMA. 1990; 7(1): 41–53.

[22] Brown JS, Parman AO. Consequences of size-selective harvesting as an evolutionary game In: The Exploitation of Evolving Resources. Springer; 1993 p. 248–261.

[23] Eikeset AM, Richter A, Dunlop ES, Dieckmann U, Stenseth NC. Economic repercussions of fisheries-induced evolution. Proceedings of the National Academy of Sciences. 2013;110(30): 12259–12264. 10.1073/pnas.1212593110 PMC3725113 23836660

[24] Zimmermann F, Jørgensen C. Bioeconomic consequences of fishing-induced evolution: a model predicts limited impact on net present value. Canadian Journal of Fisheries and Aquatic Sciences. 2015;72(4): 612–624. 10.1139/cjfas-2014-0006

[25] Faig A. *Genetics and Fisheries Management*. University of California, Davis 2016.

[26] Hofbauer J, Sigmund K, et al Evolutionary games and population dynamics. Cambridge University Press; 1998.

[27] Vincent TL, Brown JS. Evolutionary game theory, natural selection, and Darwinian dynamics. Cambridge University Press; 2005.

[28] Ashley MV, Willson MF, Pergams OR, O’Dowd DJ, Gende SM, Brown JS. Evolutionarily enlightened management. Biological Conservation. 2003;111(2): 115–123. 10.1016/S0006-3207(02)00279-3

[29] Salvioli M. Game theory for improving medical decisions and managing biological systems. PhD thesis. 2020; Politecnico di Milano, Milano, Italy.

[30] Ricker WE. Stock and recruitment. Journal of the Fisheries Board of Canada. 1954;11(5): 559–623. 10.1139/f54-039

[31] Perissi I, Bardi U, El Asmar T, Lavacchi A. Dynamic patterns of overexploitation in fisheries. Ecological modelling. 2017; 359: 285–292. 10.1016/j.ecolmodel.2017.06.009 28900312 PMC5569600

[32] Memarzadeh M, Britten GL, Worm B, Boettiger C. Rebuilding global fisheries under uncertainty. Proceedings of the National Academy of Sciences. 2019; 116(32): 15985–15990. 10.1073/pnas.1902657116 PMC6689946 31332004

[33] Allendorf FW, England PR, Luikart G, Ritchie PA, Ryman N. Genetic effects of harvest on wild animal populations. Trends in ecology & evolution. 2008; 23(6): 327–337. 10.1016/j.tree.2008.02.008 18439706

[34] Enberg K, Jørgensen C. Conclusion that fishing-induced evolution is negligible follows from model assumptions. Proceedings of the National Academy of Sciences. 2017;114(22): E4321–E4321. 10.1073/pnas.1700708114 PMC5465923 28536202

[35] Law R, Plank MJ. Balanced harvesting could reduce fisheries-induced evolution. Fish and Fisheries. 2018; 19(6): 1078–1091. 10.1111/faf.12313

[36] Darimont CT, Carlson SM, Kinnison MT, Paquet PC, Reimchen TE, Wilmers CC. Human predators outpace other agents of trait change in the wild. Proceedings of the National Academy of Sciences. 2009; 106(3): 952–954. 10.1073/pnas.0809235106 PMC2630061 19139415

[37] Conover DO, Munch SB. Sustaining fisheries yields over evolutionary time scales. Science. 2002; 297(5578): 94–96. 10.1126/science.1074085 12098697

[38] Swain DP, Sinclair AF, Mark Hanson J. Evolutionary response to size-selective mortality in an exploited fish population. Proceedings of the Royal Society B: Biological Sciences. 2007; 274(1613): 1015–1022. 10.1098/rspb.2006.0275 PMC2124474 17264058

[39] Sharpe DM, Hendry AP. SYNTHESIS: life history change in commercially exploited fish stocks: an analysis of trends across studies. Evolutionary Applications. 2009; 2(3): 260–275. 10.1111/j.1752-4571.2009.00080.x 25567879 PMC3352497

[40] Devine JA, Wright PJ, Pardoe HE, Heino M. Comparing rates of contemporary evolution in life-history traits for exploited fish stocks. Canadian Journal of Fisheries and Aquatic Sciences. 2012; 69(6): 1105–1120. 10.1139/f2012-047

[41] Palumbi SR. Humans as the world’s greatest evolutionary force. Science. 2001; 293(5536): 1786–1790. 10.1126/science.293.5536.1786 11546863

[42] Law R. Fisheries-induced evolution: present status and future directions. Marine Ecology Progress Series. 2007; 335: 271–277. 10.3354/meps335271

[43] Browman HI, Law R, Marshall CT. The role of fisheries-induced evolution. Science. 2008; 320(5872): 47–50. 10.1126/science.320.5872.47b 18388275

[44] Hairston NG Jr, Ellner SP, Geber MA, Yoshida T, Fox JA. Rapid evolution and the convergence of ecological and evolutionary time. Ecology Letters. 2005; 8(10): 1114–1127. 10.1111/j.1461-0248.2005.00812.x

[45] De Roos AM, Boukal DS, Persson L. Evolutionary regime shifts in age and size at maturation of exploited fish stocks. Proceedings of the Royal Society B: Biological Sciences. 2006; 273(1596): 1873–1880. 10.1098/rspb.2006.3518 PMC1634766 16822746

[46] Coltman DW. Evolutionary rebound from selective harvesting. Trends in Ecology & Evolution. 2008;23(3): 117–118. 10.1016/j.tree.2007.12.002 18261825

[47] Olsen EM, Heino M, Lilly GR, Morgan MJ, Brattey J, Ernande B, et al Maturation trends indicative of rapid evolution preceded the collapse of northern cod. Nature. 2004; 428(6986): 932 10.1038/nature02430 15118724

[48] Ernande B, Dieckmann U, Heino M. Adaptive changes in harvested populations: plasticity and evolution of age and size at maturation. Proceedings of the Royal Society of London Series B: Biological Sciences. 2004; 271(1537): 415–423. 10.1098/rspb.2003.2519 15101701 PMC1691608

[49] Laugen AT, Engelhard GH, Whitlock R, Arlinghaus R, Dankel DJ, Dunlop ES, et al Evolutionary impact assessment: accounting for evolutionary consequences of fishing in an ecosystem approach to fisheries management. Fish and Fisheries. 2014; 15(1): 65–96. 10.1111/faf.12007 26430388 PMC4579828

[50] Hutchings JA, Kuparinen A. Implications of fisheries-induced evolution for population recovery: Refocusing the science and refining its communication. Fish and Fisheries. 2019; 21(2): 453–464. 10.1111/faf.12424

[51] Zimmermann F, Jørgensen C. Taking animal breeding into the wild: Regulation of fishing gear can make fish stocks evolve higher productivity. Marine Ecology Progress Series. 2017; 563: 185–195. 10.3354/meps11996

[52] Diekert FK, Hjermann DØ, Nævdal E, Stenseth NC Spare the young fish: optimal harvesting policies for North-East Arctic cod Environmental and Resource Economics 2010; 47(4): 455–475. 10.1007/s10640-010-9388-z

[53] Andersen KH, Brander K. Expected rate of fisheries-induced evolution is slow. Proceedings of the National Academy of Sciences. 2009; 106(28): 11657–11660 10.1073/pnas.0901690106 19564596 PMC2710621

[54] Heikinheimo O, Setälä J, Saarni K, Raitaniemi J. Impacts of mesh-size regulation of gillnets on the pikeperch fisheries in the Archipelago Sea, Finland. Fisheries Research. 2006; 77(2): 192–199. 10.1016/j.fishres.2005.11.005

[55] Olsder G. Phenomena in inverse Stackelberg games, part 1: Static problems. Journal of Optimization Theory and Applications. 2009; 143(3): 589 10.1007/s10957-009-9572-x

[56] Olsder G. Phenomena in inverse Stackelberg games, part 2: dynamic problems. Journal of Optimization Theory and Applications. 2009; 143(3): 601 10.1007/s10957-009-9572-x

[57] Pinsky ML. Throwing back the big ones saves a fishery from hot water. Proceedings of the National Academy of Sciences. 2018; 115(8): 1678–1680. 10.1073/pnas.1722620115 PMC5828640 29440438

[58] Birkeland C, Dayton PK. The importance in fishery management of leaving the big ones. Trends in Ecology & Evolution. 2005; 20(7): 356–358. 10.1016/j.tree.2005.03.015 16701393

[59] Knapp G, Murphy JJ. Voluntary approaches to transitioning from competitive fisheries to rights-based management: bringing the field into the lab. Agric. Resour. Econ. Rev. 2010; 39(2): 213–226. 10.1017/S1068280500007279

[60] Jusup M, Maciel-Cardoso F, Gracia-Lázaro C, Liu C, Wang Z, Moreno Y. Behavioural patterns behind the demise of the commons across different cultures. Royal Society open science. 2020; 7(7): 201026 10.1098/rsos.201026 32874666 PMC7428227

[61] Cardenas JC, Janssen M, Bousquet F. Dynamics of rules and resources: three new field experiments on water, forests and fisheries. Handbook on experimental economics and the environment. 2013 10.4337/9781781009079.00020

[62] Schill C, Lindahl T, Crépin AS. Collective action and the risk of ecosystem regime shifts: insights from a laboratory experiment. Ecology and Society. 2015; 20(1) 10.5751/ES-07318-200148

[63] Kimbrough EO, Wilson BJ. Insiders, outsiders, and the adaptability of informal rules to ecological shocks. Ecological Economics. 2013; 90: 29–40. 10.1016/j.ecolecon.2013.02.008

[64] Brown JS, Staňková K. Game theory as a conceptual framework for managing insect pests. Current Opinion in Insect Science. 2017; 21: 26–32. 10.1016/j.cois.2017.05.007 28822485

[65] Staňková K, Brown JS, Dalton WS, Gatenby RA. Optimizing cancer treatment using game theory: A review. JAMA Oncology. 2019; 5(1): 96–103. 10.1001/jamaoncol.2018.3395 30098166 PMC6947530

