# Supplementary material for: Fisheries management as a Stackelberg Evolutionary Game: Finding an evolutionarily enlightened strategy
Source: PLoS One. 2021 Jan 20;16(1):e0245255. doi: 10.1371/journal.pone.0245255 (PMC7817040; doi:10.1371/journal.pone.0245255)
Supplement: S1 Appendix — (PDF) [file pone.0245255.s001.pdf]

S1 Appendix

Concavity analysis

The profit function for the domestication approach is concave with respect to H, u and z, respectively, as it can be also seen in Fig S1.

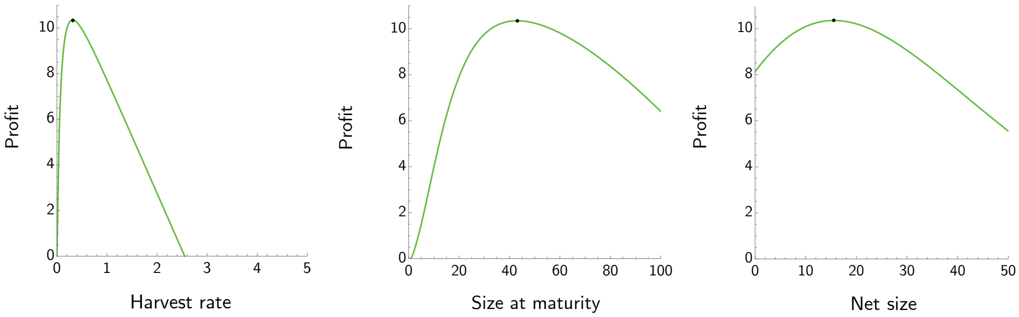

Fig. S1. The profit for the domestication approach. For each subplot we assume that the manager can control two variables and maximizes the profit function with respect to them for different values of the third variable. In the first subplot, the managers can control u and z and maximize the profit with respect to H; in the second, they can control H and z and maximize the profit with respect to u; in the third, they can control H and u and maximize the profit with respect to z. The profit function is concave with respect to H, u and z.

Evaluating the equilibria

The ecologically enlightened manager

The ecologically enlightened manager's optimal harvesting rate is a function of the fishes' size strategy, given by $\frac{\partial Q}{\partial H} = 0$.

$$\frac{\partial Q}{\partial H} = 0 \iff \frac{\partial}{\partial H}\left(\frac{HR}{u(H+d)}\log\left(\frac{sPuM}{H+d}\right)\left(\frac{z(H+d)+g}{PMe^{d\tau}} - g\right) - cH\right) = 0 \iff$$

$$\frac{R}{u(H+d)gPMe^{dT}}\left[\log\left(\frac{sPuM}{H+d}\right)\left(dgz + \frac{d\,g^2(1-PMe^{d\tau})}{H+d} + Huz(H+d) + Hug - Hz^2(H+d)\right) + \right.$$

$$\left. -H\left(uz(H+d)+ug-z^2(H+d)\right) - \frac{H}{H+d}g^2(1-PMe^{d\tau}) + Hg(u-z)PMe^{d\tau}\right] - c = 0.$$

To determine the ESS value for the population's adult size we need to determine when $H < \frac{g}{z} - d$. This will occur when $\frac{\partial Q}{\partial H}$ evaluated at $H = \frac{g}{z} - d$ and $u = z$ is negative. Evaluating $\frac{\partial Q}{\partial H}$ at $H = \frac{g}{z} - d$ and $u = z$ gives:

$$\frac{\partial Q}{\partial H}\Big|_{H+d=\frac{g}{z};u=z} = \frac{R}{g^2}\left[zgd\log\frac{sz^2M}{g} + zg\left(\frac{g}{z} - d\right)\left(\log\frac{sz^2M}{g} - 1\right)\right] - c < 0 \iff$$

$$\frac{R}{g^2}\Big[g^2\log\frac{sz^2M}{g}+g(zd-g)\Big]-c<0 \iff \log\frac{sz^2M}{g}<1+\frac{c}{R}-\frac{zd}{g}\iff z^2<\frac{g}{s}e^{1+\frac{c}{R}}.$$

The evolutionary enlightened manager

When $u = g/(H+d)$, the necessary condition for the optimal harvesting rate H_{EVO} is found by substituting $g/(H+d)$ for u in Eq 1, taking the derivative with respect to H and setting it equal to zero.

$$Q(u=\frac{g}{H+d},H,z)=\frac{HR}{g}\log\Big(\frac{sge^{-1+\frac{Hz}{g}}}{(H+d)^2}\Big)\Big(\frac{z(H+d)+g}{e^{-1+\frac{z(H+d)}{g}}}-g\Big)-cH \qquad \text{(S1)}$$

$$\frac{\partial Q}{\partial H}=0\iff \frac{R}{g}\log\Big(\frac{sge^{-1+\frac{Hz}{g}}}{(H+d)^2}\Big)\Big(\frac{z(H+d)+g}{e^{-1+\frac{z(H+d)}{g}}}-g\Big)+$$
$$+\frac{HR}{g}\frac{\frac{z}{g}(H+d)-2}{H+d}\Big(\frac{z(H+d)+g}{e^{-1+\frac{z(H+d)}{g}}}-g\Big)+\frac{HR}{g}\log\Big(\frac{sge^{-1+\frac{Hz}{g}}}{(H+d)^2}\Big)\frac{z-[z(H+d)+g]\frac{z}{g}}{e^{-1+\frac{z(H+d)}{g}}}-c=0\iff$$

$$\frac{R}{g}\Big[\Big(\log\Big(\frac{sge^{-1+\frac{Hz}{g}}}{(H+d)^2}\Big)-1\Big)\frac{gz(H+d)+g^2(1-e^{-1+\frac{z(H+d)}{g}})-z^2H(H+d)}{ge^{-1+\frac{z(H+d)}{g}}}+$$
$$+\frac{-Hzge^{-1+\frac{z(H+d)}{g}}-\frac{H}{H+d}g^2(1-e^{-1+\frac{z(H+d)}{g}})+dgz+\frac{dg^2}{H+d}(1-e^{-1+\frac{z(H+d)}{g}})}{ge^{-1+\frac{z(H+d)}{g}}}\Big]-c=0$$

Evaluating it at $H+d=\frac{g}{z}$:

$$\frac{R}{g}\Big[zd\Big(\log\Big(\frac{sz^2e^{-\frac{zd}{g}}}{g}\Big)-1\Big)-g+2zd\Big]-c,$$

which can be negative or positive, depending on the value of z. With our set of parameters ($s=1$, $g=5$, $R=4$, $d=0.2$, $c=5$) this derivative is always negative for $z\le 13.8$. Thus, the optimal harvesting rate H_{EVO} will always be lower than $g/z-d$, and, consequently, the fish size will always be equal to $g/(H+d)$ when $z\le 13.8$. When $z>13.8$, $H=g/z-d$, $u=z$ and the evolutionarily enlightened approach will coincide with the one of the ecologically enlightened manager.

Stability of the equilibria

In this subsection we give a straightforward derivation of the equation governing the fish population and determine the stability of the equilibrium solutions of (S2) as a function of the parameters u and H. This is essential in order to determine whether the equilibrium solutions found for the different strategies indeed give rise to a stable fish population.

To investigate whether the different strategies will lead to feasible and stable values for the fish population, that is stable N^*, we investigate the linear stability of the equilibrium N^*. The adult fish population of size N_t is governed by the equation

$$\frac{\mathrm{d}\,N_t}{\mathrm{d}\,t}=suP(u)M(u)N_{t-T}e^{-uN_{t-T}/R}-(H'+d)N_t, \qquad \text{(S2)}$$

where s is the fecundity rate, d the natural death rate, and R determines the effect of the surroundings on the growth of the fish and $M(u)=e^{-du/g}$ models the natural

population mortality, $H'(u) = H$ for $u \geq z$ and $H'(u) = 0$ for $u < z$. If we assume here that the individual fish chooses the same strategy as all the other fish, Eq (S2) can be derived as follows. Assume that we have two populations: a population of juveniles and an adult population of fish. The assumption that the juveniles grow linearly in time with growth rate g leads to a partial differential equation for the distribution of juveniles $j(v,t)$ of size v at time t given as

$$\frac{\partial j}{\partial t} + g\frac{\partial j}{\partial v} = -(H' + d)j, \tag{S3}$$

with boundary condition

$$j(v = 0, t) = \frac{su}{g} N_t e^{-N_t u/R},$$

which accounts for $suN_t e^{-N_t u/R}$ neonates being spawned at time t, where u is now the size of an adult fish. If we solve Eq (S3) and use $T = u/g$, we obtain:

$$j(v,t) = \frac{su}{g} N_{t-T} e^{-uN_{t-T}/R} e^{-H'(v)(v-z)/g} e^{-dv/g}. \tag{S4}$$

The adult fish now evolve according to

$$\frac{\mathrm{d}\, N_t}{\mathrm{d}t} = gj(u,t) - (H'(u) + d)N_t, \tag{S5}$$

where the contribution $j(u,t)$ to the growth of N_t is the number of juveniles promoted to adults of size $u = gT$ and the loss is due to fishing H' and natural death d. The stationary state of Eq (S2) satisfies

$$suP(u)M(u)Ne^{-uN/R} = (H + d)N.$$

This equation has the trivial solution $N_{\text{triv}} = 0$ and a non-vanishing solution

$$N^* = \frac{R}{u} \log\left(\frac{suPM}{H + d}\right). \tag{S6}$$

We can easily calculate the linear stability of the solutions. The linear stability of N_{triv} is found by linearizing Eq (S2) around 0: $N(t) = \delta N(t) + 0$, which yields

$$\frac{d\delta N}{dt} = suP(u)M(u)\delta N(t - T) - (H + d)\delta N(t). \tag{S7}$$

Substitution of $\delta N(t) = \epsilon e^{at}$, where ϵ is a small number, gives up to linear order in ϵ

$$a = suP(u)M(u)e^{-aT} - (H + d), \tag{S8}$$

from which we find the condition for stability for $N = 0$

$$H + d > suP(u)M(u). \tag{S9}$$

We remark that this condition coincides with the condition for N^* to be negative. Hence as soon as a nontrivial solution for N exists, the trivial solution turns unstable. Performing a similar analysis on the N^* solution leads to the following equation in terms of a

$$1 + \frac{a}{H + d} = e^{-aT}\left[1 - \log\left(\frac{suP(u)M(u)}{H + d}\right)\right].$$

This equation has only real solutions for a if $a < 0$, implying stability of N^* to all in phase perturbations. For out of phase perturbations we consider complex solutions of

the form $a = a_R + ia_I$. Substitution of $a = a_R + ia_I$ in $\delta N(t) = \epsilon e^{at}$, with ϵ a small parameter yields the following two equations

$$\begin{aligned} 1 + \frac{a_R}{H+d} &= e^{-a_R T}\cos(a_I T)\left[1 - \log\left(\frac{suP(u)M(u)}{H+d}\right)\right] \\ \frac{a_I}{H+d} &= -e^{-a_R T}\sin(a_I T)\left[1 - \log\left(\frac{suP(u)M(u)}{H+d}\right)\right], \end{aligned} \tag{S10}$$

from which a_I can be solved in terms of a_R,

$$\frac{a_I}{H+d} = \sqrt{e^{-2a_R T}\left(1 - \log\left(\frac{suP(u)M(u)}{H+d}\right)\right)^2 - \left(1 + \frac{a_R}{H+d}\right)^2}. \tag{S11}$$

By next taking the ratio of the two equations in (S10), we can find that a_R is given in terms of a_I as

$$a_R = -H - d - \frac{a_I}{\tan(a_I T)}. \tag{S12}$$

By one more substitution, namely of Eq (S11) into Eq (S12), we arrive at

$$\frac{a_R}{H+d} = -1 - \frac{\sqrt{e^{-2a_R T}\left(1 - \log\left(\frac{suP(u)M(u)}{H+d}\right)\right)^2 - \left(1 + \frac{a_R}{H+d}\right)^2}}{\tan\left(\sqrt{e^{-2a_R T}\left(1 - \log\left(\frac{suP(u)M(u)}{H+d}\right)\right)^2 - \left(1 + \frac{a_R}{H+d}\right)^2} T(H+d)\right)}. \tag{S13}$$

We refer to the right hand side of (S13) as $f(a_r)$. If Eq (S13) does not have any positive solution for a_R, the solution N^* is stable, otherwise there will be oscillatory instabilities. Let us first remark that in order for instabilities to occur it is necessary that $-1 + \log\left(\frac{suP(u)M(u)}{H+d}\right) > 1$ since otherwise the first equation of Eq (S13) will never have a solution. Next we notice that a solution of Eq (S13) is expected positive when the argument of tangent function is between $\pi/2$ and π, as then the second term as a whole is positive. We can bound the conditions for a stable equilibrium by the following inequality

$$1 + \sqrt{1 + \frac{\pi^2 g^2}{4(H+d)^2 u^2}} < \log\left(\frac{suP(u)M(u)}{H+d}\right) < 1 + \sqrt{1 + \frac{\pi^2 g^2}{(H+d)^2 u^2}} \tag{S14}$$

Of course, the boundary between the stable and unstable regime is determined by setting $a_R = 0$ in Eq (S14), which leads to

$$\sqrt{\left(1 - \log\left(\frac{suP(u)M(u)}{H+d}\right)\right)^2 - 1} + \tan\left(\sqrt{\left(1 - \log\left(\frac{suP(u)M(u)}{H+d}\right)\right)^2 - 1}(H+d)u/g\right) = 0. \tag{S15}$$

In Figure S2 we plot the result of the stability calculations. More insightful is Figure S3 in which we plotted the different stability regions. From Figure S4 it is clear that for small values of H ($H < 0.3$) and $u \geq 30$, the obtained solutions will not be stable. Numerical investigations show oscillatory behavior in this region. Eq (S2) is coupled to the equation of the yield rate Y, which is equal to $P + cH$, which can be controlled by

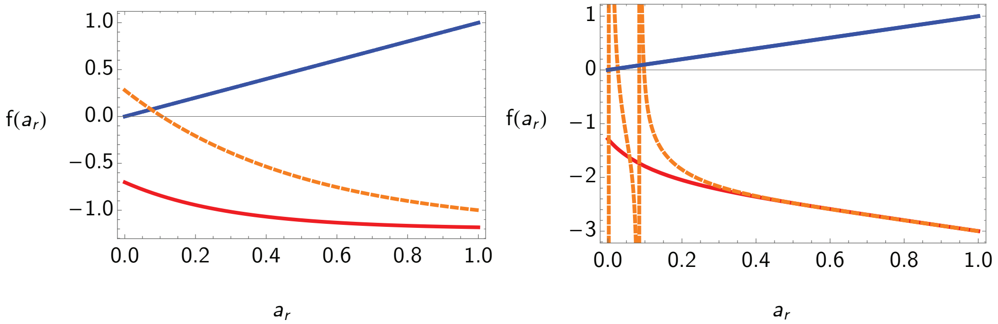

Fig. S2. In the left-hand panel we plotted the solution of Eq (S8) for two different values of u to study the stability of $N = 0$. The orange (dashed) curve corresponds to $u = 6$ and the red (solid) curve to $u = 10$. As the orange curve crossed the line $y = x$ (or rather $f(a_r) = a_r$), the trivial solution $N = 0$ is stable for $u = 10$, but unstable for $u = 6$. In the right-hand panel we plotted the stability curves for the N^* given by Eq (S13). Again the red (solid) curve (corresponding to $u = 7$) does not intersect the blue line and is therefore stable, whereas the orange curve corresponding to $u = 10$ does intersect the blue line $f(a_r) = a_r$ and is therefore an unstable solution. All other parameters where kept the same in both plots. More precisely $z = 3$, $g = 3$, $d = 0.2$, $s = 1$, and $H = 1$. Hence we find that the solutions are stable in a rather limited interval of u values $u \in [6, 10]$. Of course this also depends on the values of the other parameters.

the fisherman. The rate of biomass harvested at time t is, which also follows easily from the interpretation of the distribution of fish can be obtained as

$$\begin{aligned} Y(u,H,z,t) &= H'(u)uN_t + H'(u)\int_z^u vj(v,t)\,dv = H'(u)uN_t + \\ &+ H'(u)suN_{t-T}e^{-uN_{t-T}/R}e^{-dz/g}\left[\frac{-e^{-(H+d)(u-z)/g}[(H+d)u+g]+[(H+d)z+g]}{(H+d)^2}\right], \end{aligned} \tag{S16}$$

where we used that the total yield is the yield of the adult fish and the juveniles for the first equality and the expression for $j(v,t)$ from Eq (S4) to arrive at the second equality. When we next evaluate expression (S16) in the limit that $t \to \infty$, that is for $N_t \to N^*$, with N^* given by Eq (S6), we find the equilibrium value for Y as

$$\begin{aligned} Y_{\text{eq}}(u,H,z) &= H'(u)R\log\left(\frac{suP(u)M(u)}{H+d}\right) + H'(u)R\log\left(\frac{suP(u)M(u)}{H+d}\right)\cdot \\ &\cdot \frac{e^{-dz/g}}{uP(u)M(u)}\frac{g}{H+d}\left(-e^{-(H+d)(u-z)/g}((H+d)u/g+1)+(H+d)z/g+1\right). \end{aligned} \tag{S17}$$

Indeed Eq (S17) leads to exactly the same profit function $Q = Y - cH$ as the one given in Eq. 4. To summarize, we found in this subsection how taking the viewpoint of fish distribution leads to a natural derivation of the evolution equation for the fish population as well as for the expression for the profit Q. Moreover, we have found that the stability region for N^* stretches over a rather large part of the H values. Only for very small values of H, instability in the fish population may arise, which could lead to modified conclusions regarding the optimal strategy. In practice this would not occur, as typical the optimal values for H are sufficiently large.

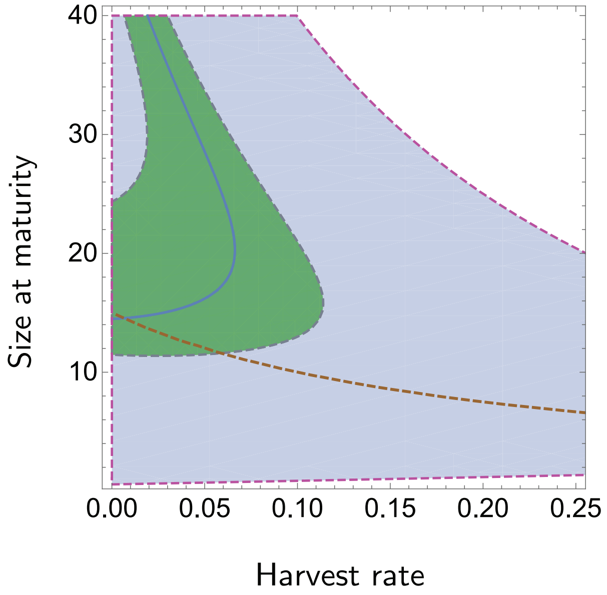

Fig. S3. The stability regions in the (H, u) plane for the parameter values $z = 3, g = 3, s = 1, d = 0.2$. The green area denotes the boundaries as given by Eq (S14). The blue curve is numerically calculated stability boundary curve following from Eq (S15). To the right of the blue curve the stationary solution is stable until the dashed magenta curve is reached, which is defined by $N^* = 0$. When H is too large the fish will go extinct and $N = 0$ is the only stable solution. The brown dashed curve is the optimal strategy curve for the fish $u = g/(H + d)$.

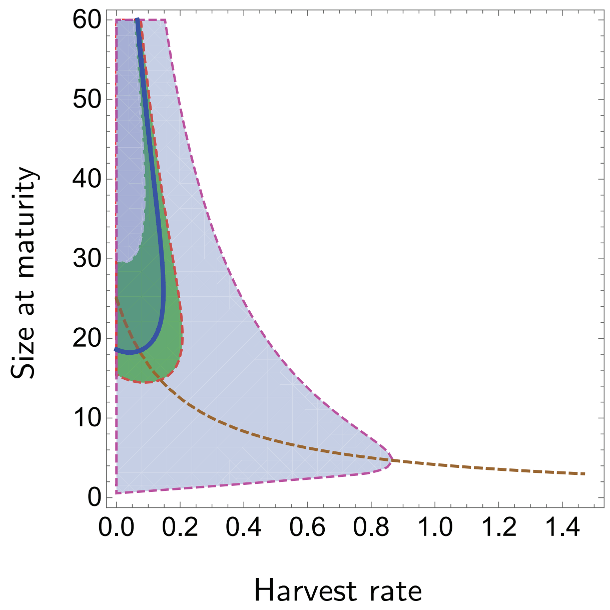

Fig. S4. The stability regions in the (H, u) plane for the parameter values $z = 3$, $g = 5$, $s = 1$, $d = 0.2$. The green area denotes the boundaries as given by Eq (S14). The blue curve is numerically calculated stability boundary curve following from Eq (S15). To the right of the blue curve the stationary solution is stable until the dashed magenta curve is reached, which is defined by $N^* = 0$. When H is too large the fish will go extinct and $N = 0$ is the only stable solution. So the stability region for u determined by $u = g/(H + d)$, is for $H \in [0.1, 0.8]$.
